# Acceptability of the use of crossword puzzles as an assessment method in Pharmacology

**DOI:** 10.30476/jamp.2021.90517.1413

**Published:** 2021-07

**Authors:** PRITHPAL SINGH MATREJA, JASPREET KAUR, LALENDRA YADAV

**Affiliations:** 1 Department of Pharmacology, TeerthankerMahaveer Medical College & Research Center, Moradabad, Uttar Pradesh – 244001, India; 2 Department of Physiology, TeerthankerMahaveer Medical College & Research Center, Moradabad, Uttar Pradesh – 244001, India

**Keywords:** Assessment, Pharmacology, Feedback

## Abstract

**Introduction:**

Didactic lectures lead to lack of attention and limited independent thinking with limited self-assessment, crossword puzzles having shown promising results and substantial usability. A thorough literature search showed that most of the studies done were on the introduction of crosswords as a tool of assessment in either medical or allied health sciences; hence, we planned this study to know the acceptability of crossword puzzles as a method of assessment.

**Methods:**

The subjects of this cross-sectional study to assess the acceptability of crossword puzzles were 5th semester students of the second professional MBBS (Bachelor of Medicine and Bachelor of Surgery). The study was conducted for three consecutive batches over a period of three months at the same time of the year from 2018 to 2020. The students had appeared for formative assessment and gave written informed consent. The crossword puzzles were given to the students, being followed by an anonymous feedback questionnaire and formative examination one week later. The feedback questionnaire was analyzed for coefficient of reliability by Cronbach’s Alpha, giving an internal consistency of 0.841.

**Results:**

A total of 425 students participated in the study. The crossword puzzles had an average percentage score of 62.7% across all the three years, which was significantly higher (p<0.05), as compared to the formative examination score of 45.2%. The same set of students appeared for both types of evaluation and the results were compared. The feedback given was that majority of the students agreed that crosswords enhanced their knowledge of drugs, promoted active learning, and helped in remembering the topic.

**Conclusion:**

Crossword puzzles were an acceptable and effective tool for assessment as they gave better results and provided better understanding in comparison to the conventional formative assessment as the feedback questionnaire showed. Most students agreed that the crosswords enhanced their knowledge of drugs, promoted active learning and helped in remembering the topic.

## Introduction

Self-learning in medical education makes learning more efficient, effective and meaningful and in this process effective time management for reinforcing skill and knowledge can be incorporated by the faculty ( 1, 2). Didactic lectures are one of the most common modes of teaching methods used in higher education institutes, making students passive listeners, and so they may lead to lack of attention and limited independent thinking with limited self-assessment ( 3- 5). There is a need to adopt and develop some modern pedagogic methodologies in Medical Education to promote critical thinking, problem solving and active learning in Medical students to enhance or supplement conventional lecture teaching ( 6). Involving students in “real life” situations fosters the learning process and provides environments that are student-centered ( 2, 3, 7). Active engagement of students with course material via problem-based learning, think-pair-share, group projects and other methods gives students a chance to reflect their own thoughts, listen to others as well as speak up and gear up to achieve objectives in the learning environment ( 2, 4, 8). This process places a greater degree of responsibility on students, though didactic lectures still are essential in guidance ( 3, 4). Incorporating active learning strategies not only improves understanding and learning, but fosters the development of critical thinking, cooperative learning, concept formation and increased motivation, as well ( 4, 9). Many methods have been adopted to supplement traditional teaching including the use of card games and puzzles in gastrointestinal physiology, panel board games in immunology, jeopardy-style game in obstetrics, and frame game in psychiatry ( 9). Crossword puzzles are one pedagogical tool that can motivate the students to know factual information and correct spellings, develop healthy skepticism and encourage logical thinking ( 6, 9). Crossword puzzles help students in critical thinking while they are pondering through various clues with narrowing the possible range of answers. These help in identifying areas with lack of comprehension and weakness, along with increase in confidence ( 3). Many forms of crossword games are used which include US style symmetrical crosswords and cryptic crosswords to name a few ( 9). Crossword puzzles provide a quick and effective method for reinforcing essential vocabulary and critical concept with a better recall, and providing appropriate discussion and humor ( 2 , 9). Crosswords have been very useful for active learning in medicine and allied areas with promising results and substantial usability ( 6).

A study done is South Africa demonstrated that crosswords made them think critically and there was a positive relationship between student collaborative learning and crosswords, where this tool greatly enhanced students engagement ( 3), and use of crosswords provided an opportunity to discuss and recall essential concepts, think critically and collaborate in small groups in Pathology, Forensic Medicine and Pharmacology ( 1, 6, 9- 11). Use of crosswords as an innovative and entertaining method was valuable to motivate students much more in the subject of Physiology ( 4). A thorough literature search showed that most of the studies done were on the introduction of crosswords as a tool of assessment in either medical or allied health sciences; hence, we planned this study to investigate the acceptability of students over subsequent batches on the use of crosswords as one of the methods for assessment in pharmacology.

## Methods

### Study setting

This cross-sectional study investigated the acceptability of a newer assessment technique/module i.e. crossword puzzles. The subjects of the study were 5th semester students of the second professional MBBS (Bachelor of Medicine and Bachelor of Surgery) course of Teerthanker Mahaveer Medical College, Moradabad, West Uttar Pradesh. The study was conducted for three consecutive MBBS batches over a period of three months at the same time of the year from 2018 to 2020 (between the month of August to September each year). The students, who had appeared for formative assessment, gave written informed consent. From the total of 450 students (150 students per year), a total of 425 students actively participated in this study. Nine students from the year 2018 participated for the validation of the questionnaire and hence were excluded from the study, and 11 students from the year 2019 and 5 students from the year 2020 missed one of the formative assessment methods and they were also excluded from the final analysis of the results. For the initial two years the mode of conducting crossword puzzles was offline, but during the year of 2020, due to the pandemic, the crossword puzzles were conducted online via the portals of Google form and Google classroom. 

### Study design

This cross-sectional study was conducted on Second Professional MBBS students over a period of three years for three consecutive batches. Three consecutive sessions at the same time of the year were performed and analyzed. The students who had participated in this study were evaluated with the help of formative examination and formative examination and feedback questionnaire. The Institutional Review Board approval was taken before the start of the study.

### Sensitization

Before initiating the session each year the faculty members of the department were sensitized with the concept of crossword puzzles and inputs were taken from the faculty on the feedback questionnaire. The same feedback questionnaire, developed after the consensus of the faculty, was given to the students each year. The session was followed by another session and the students were explained the procedure and timing interval for answering the crosswords. The unit selected for each batch was the same and also it contained the same number of questions i.e. a total of 32 questions on the Unit of Drugs acting on the endocrine system, with the option of 15 questions in the down section and 17 question in the across section ( 2). All the clues for the crossword were substantiated from standard textbooks of pharmacology that the MBBS students tend to follow. For validation of the feedback questionnaire a pilot study was done on 15 students who were not the part of our final result analysis. The feedback questionnaire was analyzed for coefficient of reliability by Cronbach’s Alpha and gave an internal consistency of 0.841. The 10-item student feedback questionnaire was recorded on a 5-point Likert Scale with scores of 1 for ‘Strongly Agree’ to 5 for ‘Strongly Disagree’ ( 2). 

The Topic of ‘Drugs acting on Endocrine system’ was done as per the teaching schedule of each batch at the same time every year, the mode of teaching the present system was offline with the mode of didactic lecture for the year of 2018 and 2019, while the mode of teaching the system in the year 2020 was online. The teaching was done at the same time each year from 2018 to 2020. The day of the crossword puzzles was informed to the students one month prior to its conduct. For the year 2018 and 2019 the print copies of crossword puzzle were administered to the students, but for the year 2020, due to the unprecedented situation, the crossword puzzles were conducted via online media with the help of Google Form and Classroom. The time duration for each year was the same, 1 hour. After the completion of the crossword puzzle the students were given an anonymous feedback questionnaire for response and comment which was collected later. One week later the students took a formative examination, the results of both were compared.

### Statistical Analysis

The evaluation was done by comparing student learning in both forms i.e. crossword puzzle, and feedback in the form of questionnaire. Feedback was expressed as percentage of response. The data was tabulated as mean±standard deviation (mean±SD), the results being analyzed using non parametric (Chi Square Test) and parametric (Unpaired Student ‘t’ test and ANOVA for comparison of result of all the three batches) tests. A p<0.05 was considered statistically significant. The data was analyzed using the Graph Pad Software.

### Ethical Consideration

The study was approved by Institutional Ethics Committee (ICE) and conducted in accordance with ICH-GCP guidelines.

## Results

From the total of 450 students of three batches - 25 students (9 students from the year 2018 participated for the validation of questionnaire and hence were excluded from the study, 11 from the year 2019 and 5 from the year 2020 missed one of the formative assessment methods and so they were also excluded from the final analysis of result). Therefore, a total of 425 students participated in the study and were the part of analysis of data. A total of 141 students in year 2018; 139 students in the year 2019 and 145 students in the year 2020 took part in the study and the results of these students were analyzed. The crossword puzzles had a maximum of 25 questions each carrying one mark, the average percentage score of the students across all the three years being 62.7±5.7% with a range of score between 8-84%. The score of formative assessment across all three years was 45.2±2.8% with a range of scores between 8-68%. The scores of each year are tabulated in Table 1. 

**Table1:** Score of students (Mean ±SD) in all three years (n=425)

Year (n)	Percentage Score in Crossword	Percentage Score in Formative Assessment	P*
2018 (n=141)	57.4±4.8	38.0±1.7	<0.0001
2019 (n=139)	61.6 ±5.3	44.8±2.9	<0.0001
2020 (n=145)	69.1 ±6.4	52.8 ± 3.4	0.0114

* p<0.05 as compared to formative assessment using unpaired student ‘t’ test.

**Figure 1:**
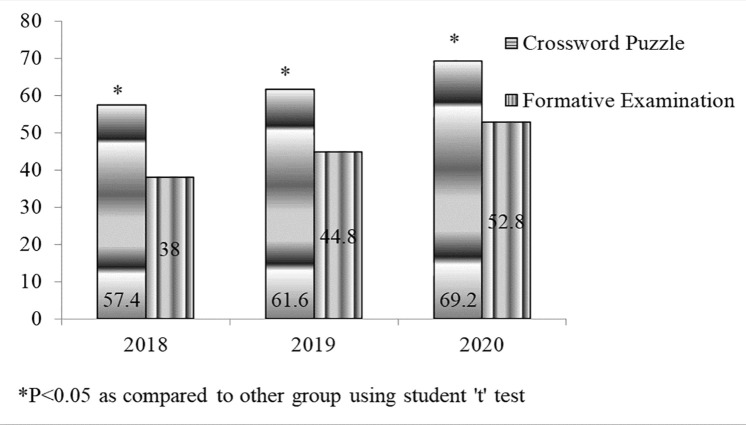
Comparison of scores of the crossword puzzle with the formative assessment

For uniformity of questions an equal proportion of weightage from each topic was chosen throughout the three years. Comparison of percentage of scores of the crossword puzzle and the formative examination is shown in Figure 1. For all the three years the scoring of students was significantly (p<0.05) higher than that of the formative assessment. 

There was a significant increase (p<0.05) in both crossword puzzle and formative examination scores as compared to previous years (Table 2).

**Table2:** Comparison of scores for all the three years

Parameters	2018	2019	2020	p*
Crossword Puzzle	57.4±4.8	61.6 ±5.3	69.1 ±6.4	0.0065
Formative Examination	38.0±1.7	44.8±2.9	52.8 ± 3.4	<0.0001

* p<0.05 using ANOVA

### Evaluation of the Feedback questionnaire 

The feedback on crossword puzzle test regarding various aspects of learning is shown in Table 3. Evaluation of the feedback questionnaire revealed a similar trend across the students of three consecutive years. Most students agreed that crosswords helped them enhance their knowledge of drugs, remember disease and drug names and increase their overall learning about the topic. 85.7 % of the students found the activity promoting active learning; 84% agreed that it promoted learning and they enjoyed faculty interaction and reviewing material while solving; and 81.2% seemed to agree with the viewpoint that it enhanced knowledge and helped in remembering the topic. The students opined that this method of assessment should be inculcated in pharmacology curriculum as a self-learning tool (Table 3). The feedback across all three years for the students showed similar pattern and there was no significant difference in the response of the students. 

**Table3:** Students feedback on crossword puzzle test regarding various aspects of learning

	Percentage of Response		
	Strongly Agree/ Agree	Neutral	Strongly Disagree/ Disagree
**Cognitive learning**			
1. Enhanced my knowledge of drugs in endocrine	81.2	10.6	8.2
2. Crossword puzzles enhanced my learning	84.0	10.4	5.6
3. Should be incorporated in pharmacology curriculum as a self-learning tool	73.6	16.0	10.4
**Affective learning**			
4. Was an enjoyable experience to solve crossword puzzles	75.3	16.5	8.2
5. The materials in the puzzles were pertinent.	80.2	14.6	5.2
6. Enjoyed faculty interaction and reviewing materials while solving	84.0	10.1	5.9
7. Length of time provided for solving the puzzles was sufficient	74.4	17.4	8.2
**Applied aspect**			
8. Helped to remember disease and drug names	81.2	12.7	6.1
9. Challenging and problem solving	67.3	22.3	10.4
10. Promoted active learning	85.7	10.1	4.2

## Discussion

The purpose of doing this study was to understand the acceptability of crosswords as a tool for assessment. The results of our study showed a similar trend across three batches as compared to the formative assessments. The students secured higher marks in the crossword puzzles. The pattern of scoring showed that there was an increase in marks secured in both types of assessment which was quite significant. As it is often presumed Pharmacology taught to second year MBBS students is quite volatile and the students are exposed to a larger amount of literature and there is often a need to incorporate an active learning method like ‘Crossword Puzzles’ to improve the understanding of the concepts of pharmacology and effectiveness in the learning process ( 2). 

Literature has shown that crosswords are an effective tool for teaching spelling, terminology, definition and giving pairing key concepts with greater retention and memorization of facts. Connecting the facts of the puzzle with clues of the word happens only when the correct spelling is known, and crossword puzzles help the students in this correct spelling a lot ( 12). 

A study done in South Africa to investigate the perception of Plickers and Crossword Puzzles for fostering active learning in 121 students demonstrated that students had the feeling that crossword puzzles gave them an opportunity to assess their understanding as well as required them to think critically and this pedagogical tool could be applied as a formative assessment instrument and offer a low-cost alternative. The results of this study are quite similar to that of ours as in our study the students also gave the feedback that crosswords helped them enhance their knowledge of drugs and promoted active learning. However, the results of our study are also different as we took students across three years and did not use plickers ( 3).

Another study done in Saskatoon, Canada to determine perception of students on usefulness of crosswords as an effective tool to reinforce essential concepts and vocabulary in Pathology showed that the students found crosswords useful and practical. The study also expressed an interest in using them as an adjunct tool. It gave an opportunity to recall essential concepts, collaborate and think critically. Our study also demonstrated that it enhanced their knowledge and promoted active learning and this method could be inculcated in the assessment. However, our study also compared the assessment of students over a period of three years which showed significant improvement in marks of crosswords over three consecutive years ( 9, 13). 

One more study done in Mumbai to study the effectiveness of crossword puzzles as an adjunct for active learning and critical thinking in Pharmacology showed significant improvement in MCQ scores in a group who had taken crossword puzzles and could be used as a supplementary educational tool in pharmacology for problem solving skills. The results of our study also showed a significant improvement in crossword puzzle scores, but we used crosswords as a means for assessment over a period of three years and they showed their acceptability ( 6).

Another Study done on First year MBBS students in UAE to assess the utility of crossword puzzles as an adjunct to lecture for physiology showed that this innovative and simulative technique was valuable for creating the interest among the students. The results of our study are similar to that of this study as in our study the students also agreed that crossword puzzles enhanced learning and promoted active learning, though our study also compared it as a mode of assessment of students over a period of three years ( 4). 

Preparing a crossword puzzle is time-consuming, but the implementation of this means of assessment is easy; it can be done using hard copies as done for initial two years in our study as well as electronically as we did for the year 2020 due to the prevailing situation. Crossword puzzles offer a certain advantage that they promote active learning and make the student go through clues, recall and review the materials. This activity leads to better retention and promotes an active learning process. The effectiveness of crossword puzzles and their acceptability by students favor their usefulness as a recreational self-learning tool in Pharmacology ( 2, 13).

There are certain limitations of our study. Firstly, due to the prevailing situation, we had to use both offline as well as online portal for assessment of both crossword and formative assessment. A uniform method for implementation might have had different results. Secondly, the lack of appropriate time as the schedule and syllabus are tightly packed, we could not implement it to all the units of Pharmacology. 

## Conclusion

To conclude, our study demonstrated that crossword puzzles were an effective tool for assessment of students and gave better results as compared to conventional formative assessment. This is shown with an improvement in scores of crossword puzzle. The feedback provided by the participants revealed a similar trend across students of three consecutive years with majority of the students agreed to the statements that it helped them enhance their knowledge of drugs, remember disease and drug names, increase overall learning about the topic, and promote active learning. The students enjoyed faculty interaction and reviewing material while solving. It also seemed to enhance knowledge and help in remembering the topic. 
